# Breathomics Detect the Cardiovascular Disease: Delusion or Dilution of the Metabolomic Signature

**DOI:** 10.2174/011573403X283768240124065853

**Published:** 2024-02-02

**Authors:** Basheer Marzoog

**Affiliations:** 1 World-Class Research Center, Digital Biodesign and Personalized Healthcare, I.M. Sechenov First Moscow State Medical University (Sechenov University), 119991 Moscow, Russia

**Keywords:** Cardiology, breathomic, cardiovascular disease, exhaled breath analysis, volatilome, ischemic heart disease

## Abstract

Volatile organic compounds (VOCs) can be subdivided into exogenous and endogenous categories based on their origin. Analyzing the endogenous VOCs can provide insights into maintaining the internal organs' homeostasis. Despite the ongoing development and the current understanding, studies have suggested a link between cardiovascular metabolic alterations in patients with ischemic heart disease and elevated levels of ethane and isoprene detectable through exhaled breath analysis. Conversely, patients with chronic heart failure exhibit elevated acetone and pentane in their exhaled air. These substances originate from disturbances in the heart tissue, including cellular and subcellular modulations. Hypothetically, ethane levels in the exhaled breath analysis can demonstrate the severity of ischemic heart disease and, consequently, the risk of death in the next 10 years due to cardiovascular disease (CVD). Real-time direct mass spectrometry is the preferred method for assessing VOCs in exhaled breath analysis. The accuracy of this analysis depends on several factors, including the selection of the relevant breath fraction, the type of breath collection container (if used), and the pre-concentration technique.

## 1 INTRODUCTION

Harnessing exhaled breath as a biomarker of the health of the organism holds immense promise as a multifaceted strategy for diagnosis, treatment, prevention, and prognosis assessment [1]. Exhaled breath harbors a diverse array of volatile organic compounds (VOCs) that serve as biomarkers of overall health, including cardiovascular health [1]. The composition of exhaled air dynamically reflects the physiological state of various bodily systems and organs. Changes in its components are known to occur in specific disease states, including gastrointestinal and pulmonary pathologies. Based on this premise, we hypothesize that individuals with cardiovascular disease exhibit distinct alterations in their exhaled air profile. Furthermore, we propose that the levels of VOCs within exhaled air may correlate with the 10-year cardiovascular mortality risk assessed using the European Society of Cardiology's SCORE2 risk evaluation tool [2].

Recently, we have seen the development of various sophisticated mass spectrometry techniques specifically tailored to accurately analyze the complex mixture of volatile compounds found in exhaled breath. These advances go beyond simple measurement of isotope abundances, allowing for high-resolution discrimination and detailed characterization of these volatile biomarkers [3].

Exhaled air analysis has been performed in patients with different pathologies including chronic obstructive lung disease, cancer, asthma, lung cancer, diabetes, arthritis, heart failure, gastric cancer, chronic kidney disease, colorectal cancer, hepatocellular carcinoma, malignant pleural mesothelioma, bladder cancer, pancreatic ductal adenocarcinoma, gastro-oesophageal cancer, peritonitis-shock, head and neck squamous cell carcinoma, multiple sclerosis and Parkinson’s disease [4-42].

While recent advancements in technology and therapeutic strategies for cardiovascular disease hold promise, accurately pinpointing the source of specific VOCs in exhaled air remains a formidable scientific challenge. The composition of exhaled breath is influenced by a complex interplay of factors, both endogenous and exogenous. Endogenous factors encompass the overall health of the organism, including the presence of cardiovascular pathologies. However, exogenous factors such as smoking exert a significant and often confounding influence on the breath profile. For example, studies have shown that compared to non-smokers, exhaled breath from smokers contains as many as 80 additional molecules, predominantly unsaturated hydrocarbons including 29 dienes, 27 alkenes, and 3 alkynes. This highlights the critical need for sophisticated analytical techniques and robust study designs to effectively disentangle the intricate web of contributions and accurately attribute VOCs to their specific biological origins [43].

Additionally, the precision and greater chance of detection of some volatile organic compounds require special preconditions including the selection of the relevant breath fraction, the type of breath collection container (if used), and the preconcentration technique [44]. Sampling of the late expiratory breath is preferred to get a greater endogenous contribution [44]. Moreover, the breath collection containers must not induce condensation on the collected sample, potentially altering VOC composition. This necessitates the development of optimized protocols for collecting, processing, and evaluating the results of exhaled breath analysis [44].

Despite the advancements in therapeutic strategies and technologies, cardiovascular disease (CVD) remains the leading cause of mortality and morbidity worldwide [45]. Tragically, every second, one person succumbs to CVD worldwide [46]. Despite the energy deprivation caused by ischemic heart disease, myocardiocytes employ compensatory mechanisms to survive and resist ischemic damage. This involves activating specific signaling pathways that increase the necrosis threshold, allowing myocardiocytes to enter a dormant state to conserve energy. Moreover, myocardiocytes enhance autophagy, a cellular recycling process, to strengthen the cellular antioxidant defense system and further reduce energy expenditure [47-60]. To enhance cardiometabolism, exogenous applications of various activators can be employed, including glycolysis activators, Sirt1 or 3 activation (autophagy induction through NAD+ administration and deacetylation), ketone oxidation promotion, pyruvate dehydrogenase complex activation (glucose oxidation), and hexosamine biosynthesis pathway activation (O-GlcNAcylation; administration of glucosamine/glucose). These approaches have shown promising cardio-therapeutic effects [61, 62]. In contrast, inhibiting mitochondrial oxygen consumption, the malate-aspartate shuttle, mitochondrial succinate metabolism (malonate), fatty acid oxidation (CD36 inhibitors, malonyl-CoA decarboxylase inhibitors), or disrupting FOF1-ATPase dimer stability and/or weakening the association of hexokinase II or creatine kinase with mitochondria can negatively impact mitochondrial cristae structure and function [61]. Current research on using exhaled breath analysis for diagnosis, follow-up of treatment regime, early prevention, and determination of prognosis in CVD patients remains in the development phase.

## 2 EXHALED AIR CHANGES IN CARDIOVASCULAR DISEASE

Part of the components of the exhaled air originate endogenously from the cellular and subcellular level as well as exogenously from the respiratory airways. The components of exhaled air demonstrate the metabolic changes in the organism, including cardio-metabolic changes [63]. Identification of the components of the exhaled air is essential to develop criteria for the diagnosis of various pathologies [64]. Unfortunately, current preclinical and clinical studies are not sufficient to produce a clinical recommendation for a rapid, safe, and non-invasive method for diagnosis, prevention, and prognosis identification through exhaled breath analysis.

In healthy individuals, exhaled air compounds include several organic and non-organic components, N2 (78%), O_2_ (17%) and CO_2_ (4%), other (1%). Organic volatile compounds in exhaled air in healthy individuals include and are not limited to hydrocarbons (24), ketones (10), terpenes (8), heterocyclic compounds (7) and aromatic compounds (7) [65]. In addition, there are several types of aldehyde in the exhaled air of healthy individuals. A recent clinical study on 26 healthy volunteers using ion flow tube-mass spectrometry (SIFT-MS) demonstrated that in between all the types of aldehydes (C3-C10), the Propanal was the most abundant in the exhaled air of the healthy volunteers [66]. In addition, acetone and isoprene (mean 950 and 130 ppb, respectively) have been found to be the most abundant in the exhaled air of healthy people (Table **1**) [65].

## 3 EXHALED AIR IN DETERMINATION OF THE PROGNOSIS OF THE CARDIOVASCULAR DISEASE

VOCs can be assessed using several methods, including real-time mass-spectrometry (SESI-HRMS (secondary electrospray ionisation - high-resolution MS), SIFT-MS (selective ion flow tube mass spectrometry) or PTR-MS (proton transfer reaction mass spectrometry)), gas chromatography coupled with single quadrupole mass spectrometry (GC/MS) [33, 67-70]. However, the most reliable method with ultrahigh specification (due to chromatographic separation) and sensitivity (parts per trillion) is MS coupled with gas chromatography (GC / MS), which is used in an offline method [16, 71-75]. However, the offline method is not helpful in terms of sample storage and transportation, where chemical reactions (sorption effect 50% in 3-6 hours) occur in between stored molecules leading to the loss of their original features [76, 77]. Direct mass spectrometry in real time is the method of choice to assess the VOCs of exhaled breath analysis (Table **2**) [75].

Single quadrupole MS is the most common type of mass spectrometer used in GC/MS, but other types of mass spectrometers can also be used, such as time-of-flight (TOF) and ion trap.

GC/MS is the gold standard technique for many applications, but PTR-MS and SESI-HRMS offer advantages in terms of speed, sensitivity, and capability for real-time analysis.

The limitations of GC / MS are the relatively long time of a single analysis and often labor-intensive sample preparation, the fragmentation of the original molecule that reduces the research efficacy in the case of multicomponent gas mixtures that include human exhalation in real time [78, 79]. However, to overcome these limitations, a novel method has been developed by adding multidimensional gas chromatography (MDGC) to increase its resolution and lower its detection limits even further (Table **3**) [78]. The basic principle of the SIFT-MS is to ionize the Volatile organic compound from a donor of electron (H_3_O^+^, NO^+^ and O_2_+) [76].

The proton transfer reaction mass spectrometry method (PTR-MS) can detect molecules with affinities greater than 166.5 kcal/mol (H_2_O), which helps to prevent the ionization of N_2_, O_2_, and CO_2_ in the exhaled air [80]. The efficacy of the PTR-MS method in detecting various alkenes, alcohols, aldehydes, aromatics, ketones, nitriles, and sulfides has been observed [81].

In terms of cardiovascular disease, the components of exhaled air include elevation in exhaled breath acetone levels in patients with heart failure [82]. Furthermore, the results of the spectrophotometry (mean concentration of acetone is 0.6 ug/L (minimum-maximum, 0.3-1.2) showed that the acetone level is dependent on the stage of heart failure, including the involvement in heart failure and liver biochemical analysis disturbance [27]. The study results of the hazard ratio by the Cox proportional multivariate regression model confirmed that the acetone level of > 1.20 ug/L is associated with a poor prognosis in patients with heart failure with a mean left ventricle ejection fraction of 32 ± 8.6% [27]. Suggesting that a high level of exhaled breath acetone is related to right heart failure and mortality as well as the patient’s prognosis. An additional study using selected ion flow tube mass spectrometry (SIFT-MS) showed that patients with acute decompensated heart failure have an elevated pentane level in exhaled breath analysis [83].

Isoprene has been described to be related to various CVDs, which requires further investigation to confirm its correlation as well as specificity and sensitivity [84]. Biochemically, isoprene is a byproduct of cholesterol synthesis [85].

**Fig. (1):**
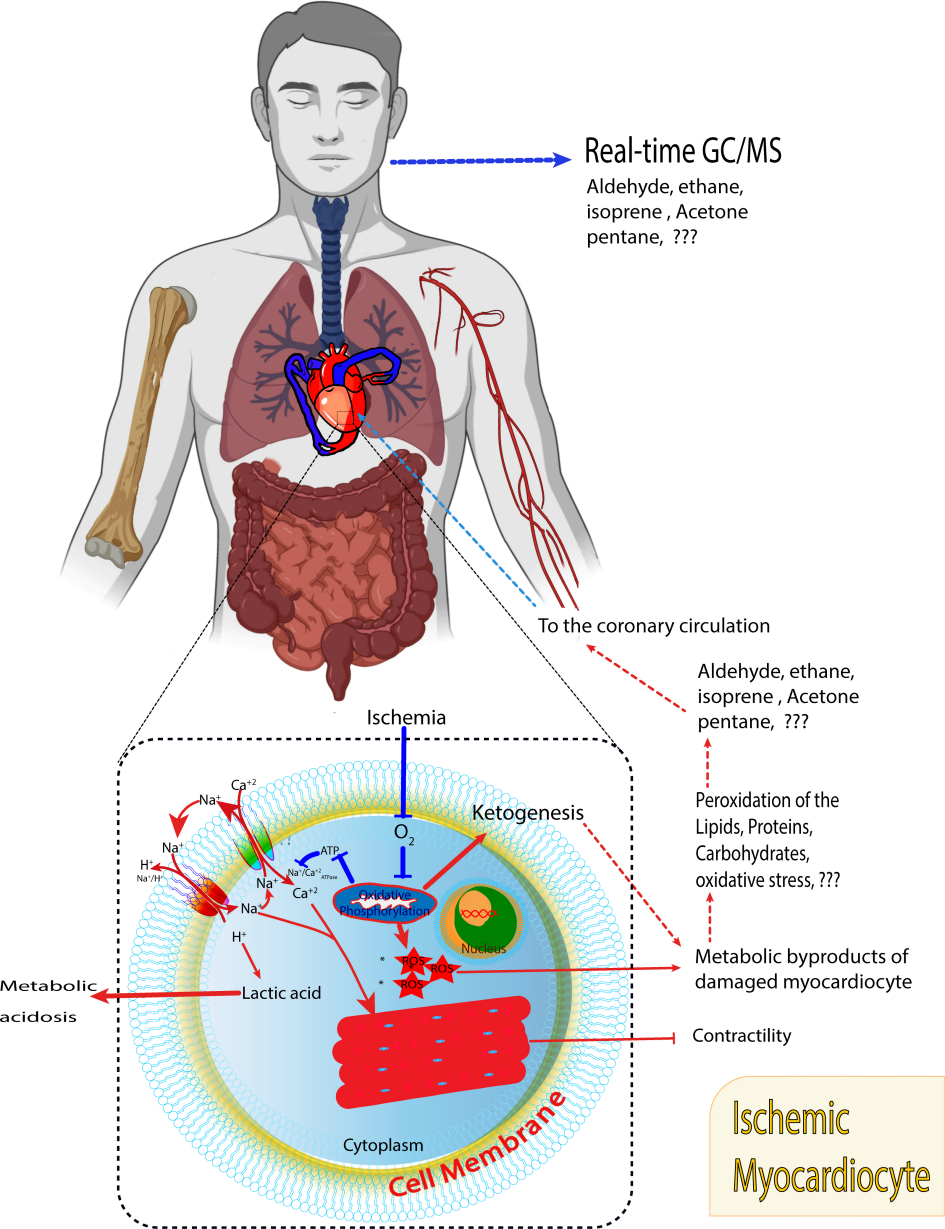
Diagram presentation of damaged myocardiocytes produces reactive oxygen species (ROS) and further organelles membrane peroxidation and cell membrane peroxidation. The lung removes the cardiometabolic byproducts from the circulation in trace amounts in form of volatile compounds that detected by the real-time GC/MS.

Additional studies in terms of ischemic heart disease; stable coronary artery disease (SCAD), post-myocardial infarction patients (post-MI), and different types of arrythmias are underestimated and require further elucidation (Fig. **1**).

## 4 EXHALED AIR ANALYSIS; CURRENT APPLICATIONS AND FUTURE CHALLENGES FOR CLINICAL IMPLICATION

The current challenges in applying exhaled breath analysis in diagnosis, treatment follow-up, prevention, and prognosis determination stem from the heterogeneity of existing exhaled breath analysis methods. This methodological diversity leads to discrepancies in exhaled breath analysis results, necessitating careful selection of sample and technique to minimize bias. The sample should come from individuals residing in a homogenous environment with well-characterized atmospheric components, and all samples should undergo analysis using a single standardized method, such as on-line gas chromatography-mass spectrometry (GC/MS) analysis of end-stage exhalations. Exhaled breath analysis has shown promising potential for detecting coronary artery disease (CAD) in patients with chest pain [86].

Classically, patients with heart failure, especially chronic variant, are associated with pulmonary inflammation which makes the conformation of the origin of the volatile organic compounds in the exhaled breath analysis [87, 88]. It is suggested that a kind of physical excretion is required to confirm the source of the compounds in the exhaled air [89]. One of the common compounds is nitric oxide (NO), which is closely related to cardiovascular disease due to the sequelae of endothelial cell dysfunction [83, 90, 91]. In cardiomyopathy patients, the NO level is observed to be elevated in the exhaled breath analysis.

The proposed diagnostic method is to improve the diagnostic accuracy of the classical physical stress methods such as bicycle ergometry in terms of ischemic heart disease detection. The physical stress test is combined with the exhaled breath analysis. The patient is asked to breathe directly into the mass spectrometer immediately after completion of the physical stress test, within a minute, and again after 3-5 minutes from the first breathing, within a minute. The patients must not clean their teeth with toothpaste or drink or eat (only water is allowed) for 6-8 hours before the breathing test. This can be performed for patients with negative, suspected, and positive results on the physical stress test to increase the sensitivity of the test in detection of the ischemic heart disease.

Several challenges need to be addressed before readily implementing the exhaled breath analysis in the diagnosis of ischemic heart disease in clinical practice. Including the heterogeneity of methods (diverse sampling and analysis techniques lead to inconsistent results, requiring standardization), data preprocessing (difficulty in analyzing and interpreting the massive amount of data from exhaled breath), source confirmation (distinguishing between lung-derived VOCs and those originating from other organs, particularly in patients with lung or blood vessel inflammation), and specificity and sensitivity (identifying reliable biomarkers specific to CVD and differentiating them from background noise).

## 5 DISCUSSION

The current obstacles that prevent using the exhaled breath analysis in clinical practice is the difficulty in analyzing the data preprocessing step of the exhaled breath [92].

The presence of VOCs in exhaled air provides evidence that the lungs play a crucial role in the body's defense mechanism by clearing toxic substances from the bloodstream. Studies using animal models have demonstrated the lung's ability to eliminate metabolic byproducts associated with various pathologies affecting different organs, including acute inflammatory pancreatitis, acute purulent pancreatitis, acute inflammatory localized and generalized peritonitis, and acute purulent localized and generalized peritonitis [93]. Thus, impairment of the lung's defense function due to congenital or infectious diseases diminishes the lungs' defense capacity, ultimately enhancing the likelihood of detecting the metabolomic byproducts of the targeted organ.

 Moreover, inflammatory pathologies of the blood vessels, including various types of vasculitis, are crucial for detecting the final metabolomic byproduct in exhaled air. This is because these products, during their transportation through the bloodstream, have the potential to interact with inflamed vessels and alter their chemical properties, leading to a distinct final metabolic byproduct in exhaled air [94]. Therefore, selecting patients who are volunteered for a clinical trial requires a full evaluation of the concomitant diseases and then progress with them.

Identification of the origin of the VOCs requires the determination of the components concentration of the room atmosphere (depending on the living place of the individual) and then the exhaled breath air compounds exhaled in healthy people [43]. Subsequently, the level of VOCs of exhaled breath in patients with CVD is assessed.

The analysis of exhaled breath air has been examined in a single prospective study to identify the last weeks of life in patients with lung cancer [7]. The results were supportive of claiming that reduction in some biomarkers and elevation in others in the exhaled air is related to the last days “dying period” of these patients [7]. Suggesting that metabolic changes in the pathways of the affected tissue are perturbated and result in releasing of these pathological substances in the circulation then to the lung and further evacuation with the air [95]. These biomarkers (pentanal, hexanal, and heptanal) originated from lipid peroxidation of the lung cancer cells [96]. However, these biomarkers vary according to the exhaling breath sampling and analysis used, where the preconcentration method is preferred in detecting unsaturated aldehydes [96]. Additionally, ethane is an additional biomarker of lipid peroxidation and has a direct correlation with the plasma lactate dehydrogenase level and C-reactive protein in patients with interstitial lung disease [97, 98]. In the context of CVD, the larger the ischemic zone, the higher the lactate dehydrogenase (LDH) level and consequently, the higher the ethane level in exhaled breath analysis. Subjecting patients with stable CAD to stress tests like treadmill or cycle ergometry can lead to impaired oxygen delivery to heart tissue and increased reactive oxygen species generation. This results in peroxidation of mitochondrial lipid membranes, organelles, and cell membranes. This, in turn, alters myocardiocyte metabolism from fatty acid beta-oxidation to glycolysis, leading to low ATP production and increased lactic acid formation (by lactate dehydrogenase). The final metabolic byproduct of lactate dehydrogenase, ethane, is detectable in exhaled breath [98]. Therefore, we propose that the ethane level in exhaled breath analysis reflects the severity of ischemic heart disease (IHD) and, consequently, the risk of death from CVD within the next 10 years. This suggests that ethane analysis has the potential for early prophylaxis of CVD, assessment of CVD risk, evaluation of IHD severity, prognostication of IHD patients, and monitoring treatment response. However, further research on patients with IHD is required to validate this hypothesis.

An ongoing clinical trial is conducted by Basheer A. Marzoog and Co-authors to examine the changes in the exhaled breath in patients with ischemic heart disease confirmed by the post-stress myocardial perfusion defect using stress myocardial perfusion computer tomography, 640 slice device with 0.5 mm thickness of the slice (NCT06181799). Additionally, the clinical trial uses special machine learning models to interpret the results of the single channel electrocardiography in the diagnosis of ischemic heart disease.

Performing a physical activity load in the form of treadmill or bicycle ergometry is to assess the fact that these volatile organic compounds most likely originated from the ischemic heart. Therefore, we suggest performing on-line GC/MS for patients with ischemic heart disease (ex. SCAD) with/ without different stages of SCAD before the physical excretion/ peak (can consider ECG depression > 1 mm as a peak) /after physical excretion (return heart rate to the original before the physical load) [99-103] and then compare the results with a control group for validation. Additionally, changes in the breathe can be used to correlate with the development of atherosclerosis in various vessels.

## CONCLUSION

Exhaled breath analysis holds promise as a multifaceted strategy for the diagnosis, treatment, prevention, and prognosis assessment of various conditions, including CVD. Exhaled air harbors a diverse array of VOCs that serve as biomarkers of overall health, including cardiovascular health. The composition of exhaled breath fluctuates in response to alterations in various body systems and organs. However, the implementation of exhaled breath analysis in clinical settings faces challenges due to the heterogeneity of existing methods. This methodological diversity leads to discrepancies in exhaled breath analysis results, highlighting the importance of careful sample selection and technique optimization to minimize bias.

A review of the literature reveals inconsistencies in reported exhaled breath analysis findings, attributable to the heterogeneity of employed methods and factors affecting the accuracy of real-time GC/MS analysis. To harmonize these results, a systematic clinical trial is warranted, featuring meticulous sample selection based on smoking status, concomitant diseases, living environment, occupation, and alcohol consumption. These factors play a crucial role in altering exhaled breath VOC profiles. Further investigations on patients with ischemic heart disease are necessary to validate the proposed hypothesis regarding exhaled breath analysis's potential for diagnosing and prognosticating CVD.

## Figures and Tables

**Table 1:** Numerous studies have investigated the potential of exhaled breath analysis to distinguish between different groups of patients with cardiovascular disease by measuring the levels of specific volatile organic compounds (VOCs).

**Study**	**VOCs Measured**	**Participants**	**Key Findings**
Exposure to Volatile Organic Compounds - Acrolein, 1,3-Butadiene, and Crotonaldehyde - is Associated with Vascular Dysfunction [100].	Alkenes, alkynes, benzene, toluene, ethyl benzene, xylene (BTEX) compounds	Not specified	Total VOC exposure associated with increased blood pressure, heart rate, and cardiovascular risk factors
Environmental exposure to volatile organic compounds is associated with endothelial injury [100].	Not specified	375 non-smoking participants with low to severe CVD risk	VOC exposure linked to deficits in endothelial repair capacity, vascular injury, and increased CVD risk
Identifying the cardiovascular effects of multiple pollutants [101]	CEMA, 3HPMA, DHBMA, MHBMA3, HPMMA	Not specified	VOC metabolites significantly associated with blood pressure, suggesting an impact on cardiovascular health
Volatile organic compounds exposure and cardiovascular effects in hair salons [102]	Not specified	Not specified	Occupational exposure to VOCs in hair salons associated with cardiovascular effects
Portable Breath-Based Volatile Organic Compound Monitoring for the Detection of COVID-19 [103]	Peak identification No. 17, 49, 91, and 94	167 participants	Four VOC biomarkers identified to distinguish between COVID-19 and non-COVID-19 patients

**Table 2:** The selection of the most appropriate mass spectrometry technique for exhaled breath analysis is contingent upon the specific requirements of the analysis, including the type of sample, the degree of sensitivity required, and the level of identification desired.

**Mass Spectrometry Technique**	**Coupling**	**Processing Steps**	**Limitations**	**Strengths**
GC/MS	Gas chromatography	Sample injection, separation in GC column, ionization (typically electron impact), mass analysis in quadrupole mass spectrometer	Limited to volatile and thermally stable compounds, complex spectra for mixtures	High sensitivity, good separation of components, well-established technique
PTR-MS	Direct sample introduction	Ionization with protonated water vapor (H_3_O^+^), mass analysis in quadrupole mass spectrometer	Limited to volatile compounds with higher proton affinity than water, interference from water vapor	Real-time analysis, high sensitivity for VOCs, simple spectra
SESI-HRMS	Direct sample introduction	Sample desolvation, ionization with corona discharge, mass analysis in high-resolution mass spectrometer	Requires line-of-sight access to sample, potential matrix effects	Excellent mass accuracy and resolution, allows identification of unknown compounds, suitable for complex samples

**Table 3:** A table summarizing the processing steps, limitations, and strengths of the multidimensional gas chromatography (MDGC).

**Processing Steps**	**Limitations**	**Strengths**
Initial sample preparation	- Time-consuming	- Efficient separation of complex mixtures
Injection and separation	- High technical expertise required	- Enhanced resolution and separation of closely related compounds
Detection	- Complex data analysis	- Sensitive detection of low-abundance compounds
Data processing and interpretation	- Specialized software required	- Comprehensive profiling of volatile organic compounds (VOCs) and their metabolites

## LIST OF ABBREVIATIONS

VOCs: Volatile Organic Compounds
CVD: Cardiovascular Disease
SESI-HRMS: Secondary Electrospray Ionisation - High-Resolution Mass Spectrometry
PTR-MS: Proton Transfer Reaction Mass Spectrometry

## References

[1] Pereira J., Porto-Figueira P., Cavaco C. (2015). Breath analysis as a potential and non-invasive frontier in disease diagnosis: an overview.. Metabolites.

[2] Hageman S, Pennells L, Ojeda F (2021). SCORE2 risk prediction algorithms: New models to estimate 10-year risk of cardiovascular disease in Europe Eur Heart J.

[3] Murray K.K., Boyd R.K., Eberlin M.N., Langley G.J., Li L., Naito Y. (2013). Definitions of terms relating to mass spectrometry (IUPAC Recommendations 2013).. Pure Appl. Chem..

[4] Trefz P., Obermeier J., Lehbrink R., Schubert J.K., Miekisch W., Fischer D.C. (2019). Exhaled volatile substances in children suffering from type 1 diabetes mellitus: Results from a cross-sectional study.. Sci. Rep..

[5] van de Kant K.D.G., van der Sande L.J.T.M., Jöbsis Q., van Schayck O.C.P., Dompeling E. (2012). Clinical use of exhaled volatile organic compounds in pulmonary diseases: A systematic review.. Respir. Res..

[6] Amal H., Leja M., Funka K. (2016). Breath testing as potential colorectal cancer screening tool.. Int. J. Cancer.

[7] Chapman E.A., Baker J., Aggarwal P. (2023). GC-MS techniques investigating potential biomarkers of dying in the last weeks with lung cancer.. Int. J. Mol. Sci..

[8] Chung J., Akter S., Han S. (2022). Diagnosis by volatile organic compounds in exhaled breath from patients with gastric and colorectal cancers.. Int. J. Mol. Sci..

[9] Sukaram T., Tansawat R., Apiparakoon T. (2022). Exhaled volatile organic compounds for diagnosis of hepatocellular carcinoma.. Sci. Rep..

[10] Politi L., Monasta L., Rigressi M.N. (2021). Discriminant profiles of volatile compounds in the alveolar air of patients with squamous cell lung cancer, lung adenocarcinoma or colon cancer.. Molecules.

[11] Di Gilio A., Catino A., Lombardi A. (2020). Breath analysis for early detection of malignant pleural mesothelioma: Volatile organic compounds (VOCs) determination and possible biochemical pathways.. Cancers.

[12] Catino A., de Gennaro G., Di Gilio A. (2019). Breath analysis: A systematic review of Volatile Organic Compounds (VOCs) in diagnostic and therapeutic management of pleural mesothelioma.. Cancers.

[13] Rodrigues D., Pinto J., Araújo A.M. (2018). Volatile metabolomic signature of bladder cancer cell lines based on gas chromatography-mass spectrometry.. Metabolomics.

[14] Princivalle A., Monasta L., Butturini G., Bassi C., Perbellini L. (2018). Pancreatic ductal adenocarcinoma can be detected by analysis of volatile organic compounds (VOCs) in alveolar air.. BMC Cancer.

[15] Chin S.T., Romano A., Doran S.L.F., Hanna G.B. (2018). Cross-platform mass spectrometry annotation in breathomics of oesophageal-gastric cancer.. Sci. Rep..

[16] Brekelmans M.P., Fens N., Brinkman P. (2016). Smelling the diagnosis: The electronic nose as diagnostic tool in inflammatory arthritis. A case-reference study.. PLoS One.

[17] DeLano F.A., Chow J., Schmid-Schönbein G.W. (2017). Volatile decay products in breath during peritonitis shock are attenuated by enteral blockade of pancreatic digestive proteases.. Shock.

[18] Krilaviciute A., Heiss J.A., Leja M., Kupcinskas J., Haick H., Brenner H. (2015). Detection of cancer through exhaled breath: A systematic review.. Oncotarget.

[19] Hanna G.B., Boshier P.R., Markar S.R., Romano A. (2019). Accuracy and methodologic challenges of volatile organic compound-based exhaled breath tests for cancer diagnosis.. JAMA Oncol..

[20] Gruber M., Tisch U., Jeries R. (2014). Analysis of exhaled breath for diagnosing head and neck squamous cell carcinoma: A feasibility study.. Br. J. Cancer.

[21] Bajtarevic A., Ager C., Pienz M. (2009). Noninvasive detection of lung cancer by analysis of exhaled breath.. BMC Cancer.

[22] Xu Z., Broza Y.Y., Ionsecu R. (2013). A nanomaterial-based breath test for distinguishing gastric cancer from benign gastric conditions.. Br. J. Cancer.

[23] Peled N., Hakim M., Bunn P.A. (2012). Non-invasive breath analysis of pulmonary nodules.. J. Thorac. Oncol..

[24] Ionescu R., Broza Y., Shaltieli H. (2011). Detection of multiple sclerosis from exhaled breath using bilayers of polycyclic aromatic hydrocarbons and single-wall carbon nanotubes.. ACS Chem. Neurosci..

[25] Buszewski B., Ligor T., Jezierski T., Wenda-Piesik A., Walczak M., Rudnicka J. (2012). Identification of volatile lung cancer markers by gas chromatography-mass spectrometry: Comparison with discrimination by canines.. Anal. Bioanal. Chem..

[26] Stott S., Broza Y.Y., Gharra A., Wang Z., Barker R.A., Haick H. (2022). The utility of breath analysis in the diagnosis and staging of parkinson’s disease.. J. Parkinsons Dis..

[27] Marcondes-Braga F.G., Gioli-Pereira L., Bernardez-Pereira S. (2020). Exhaled breath acetone for predicting cardiac and overall mortality in chronic heart failure patients.. ESC Heart Fail..

[28] Marcondes-Braga F.G., Batista G.L., Bacal F., Gutz I. (2016). Exhaled breath analysis in heart failure.. Curr. Heart Fail. Rep..

[29] Bykova A.A., Malinovskaya L.K., Chomakhidze P.S. (2019). Exhaled breath analysis in diagnostics of cardiovascular diseases.. Kardiologiia.

[30] Bykova AA, Malinovskaya LK, Trushina OV (2019). Exhaled breath analysis in diagnosis of chronic heart failure with reduced left ventricular ejection fraction.. Cardiology and cardiovascular surgery.

[31] Marcondes-Braga F.G., Batista G.L., Gutz I.G.R. (2016). Impact of exhaled breath acetone in the prognosis of patients with heart failure with reduced ejection fraction (HFrEF).. PLoS One.

[32] Malinovskaya LK, Bykova AA, Chomahidze PSH, Kopylov PHYU, Syrkin AL, Betelin VB (2018). P3758Exhaled breath analysis in the differential diagnostics of heart failure.. Eur Heart J.

[33] Biagini D., Lomonaco T., Ghimenti S. (2017). Determination of volatile organic compounds in exhaled breath of heart failure patients by needle trap micro-extraction coupled with gas chromatography-tandem mass spectrometry.. J. Breath Res..

[34] Yokokawa T., Sato T., Suzuki S. (2017). Elevated exhaled acetone concentration in stage C heart failure patients with diabetes mellitus.. BMC Cardiovasc. Disord..

[35] Yokokawa T., Sato T., Suzuki S. (2018). Change of exhaled acetone concentration levels in patients with acute decompensated heart failure a preliminary study.. Int. Heart J..

[36] Zhou Q., Wang Q., Chen B. (2019). Factors influencing breath analysis results in patients with diabetes mellitus.. J. Breath Res..

[37] Broza Y.Y., Khatib S., Gharra A. (2019). Screening for gastric cancer using exhaled breath samples.. Br. J. Surg..

[38] Wang M.H., Yuk-Fai Lau S., Chong K.C. (2017). Estimation of clinical parameters of chronic kidney disease by exhaled breath full-scan mass spectrometry data and iterative PCA with intensity screening algorithm.. J. Breath Res..

[39] Zeng Q., Li P., Cai Y. (2016). Detection of creatinine in exhaled breath of humans with chronic kidney disease by extractive electrospray ionization mass spectrometry.. J. Breath Res..

[40] Badjagbo K. (2012). Exhaled breath analysis for early cancer detection: principle and progress in direct mass spectrometry techniques.. Clin. Chem. Lab. Med..

[41] Chan M.J., Li Y.J., Wu C.C. (2020). Breath ammonia is a useful biomarker predicting kidney function in chronic kidney disease patients.. Biomedicines.

[42] Rodríguez-Aguilar M., Ramírez-García S., Ilizaliturri-Hernández C. (2019). Ultrafast gas chromatography coupled to electronic nose to identify volatile biomarkers in exhaled breath from chronic obstructive pulmonary disease patients: A pilot study.. Biomed. Chromatogr..

[43] Filipiak W., Ruzsanyi V., Mochalski P. (2012). Dependence of exhaled breath composition on exogenous factors, smoking habits and exposure to air pollutants.. J. Breath Res..

[44] Lawal O., Ahmed W.M., Nijsen T.M.E., Goodacre R., Fowler S.J. (2017). Exhaled breath analysis: A review of ‘breath-taking’ methods for off-line analysis.. Metabolomics.

[45] Tsao C.W., Aday A.W., Almarzooq Z.I. (2023). Heart disease and stroke statistics—2023 update: A report from the american heart association.. Circulation.

[46] Cardiovascular diseases (CVDs). https://www.who.int/news-room/fact-sheets/detail/cardiovascular-diseases-(cvds).

[47] Marzoog B.A., Vlasova T.I. (2022). Myocardiocyte autophagy in the context of myocardiocytes regeneration: A potential novel therapeutic strategy.. Egypt. J. Med. Hum. Genet..

[48] Marzoog B.A. (2023). Autophagy behavior in post-myocardial infarction injury.. Cardiovasc. Hematol. Disord. Drug Targets.

[49] Marzoog BA (2024). Drug Targets.

[50] Abdullah Marzoog B. (2023). Pathophysiology of cardiac cell injury in post-covid-19 syndrome.. Emir. Med. J..

[51] Marzoog B.A. (2022). Systemic and local hypothermia in the context of cell regeneration.. Cryo Lett..

[52] Marzoog B.A. (2023). Tree of life: Endothelial cell in norm and disease, the good guy is a partner in crime!. Anat. Cell Biol..

[53] Abdullah Marzoog B. (2023). Cell physiological behavior in the context of local hypothermia.. Emir. Med. J..

[54] Abdullah Marzoog B. (2023). Autophagy behavior under local hypothermia in myocardiocytes injury.. Cardiovasc. Hematol. Agents Med. Chem..

[55] Marzoog B.A. (2023). Review article: Autophagy behavior in endothelial cell regeneration.. Curr. Aging Sci..

[56] Marzoog B.A. (2023). Endothelial dysfunction under the scope of arterial hypertension, coronary heart disease, and diabetes mellitus using the angioscan.. Cardiovasc. Hematol. Agents Med. Chem..

[57] Abdullah Marzoog B. (2023). Adaptive and compensatory mechanisms of the cardiovascular system and disease risk factors in young males and females.. Emir. Med. J..

[58] Marzoog B.A. (2022). Autophagy in endothrlial cell dysfunction.. Curr. Mol. Med..

[59] Marzoog B.A., Vlasova T.I. (2021). Membrane lipids under norm and pathology.. Eur J Clin Exp Med.

[60] Abdullah Marzoog B. (2023). Caveolae’s behavior in norm and pathology.. Emir. Med. J..

[61] Zuurbier C.J., Bertrand L., Beauloye C.R. (2020). Cardiac metabolism as a driver and therapeutic target of myocardial infarction.. J. Cell. Mol. Med..

[62] Marzoog B.A., Averina D. (2023). Nicotinamide mononucleotide in the context of myocardiocyte longevity.. Curr. Aging Sci..

[63] Gaugg M.T., Nussbaumer-Ochsner Y., Bregy L. (2019). Real-time breath analysis reveals specific metabolic signatures of COPD exacerbations.. Chest.

[64] Buszewski B., Kęsy M., Ligor T., Amann A. (2007). Human exhaled air analytics: Biomarkers of diseases.. Biomed. Chromatogr..

[65] Mochalski P., King J., Klieber M. (2013). Blood and breath levels of selected volatile organic compounds in healthy volunteers.. Analyst.

[66] Huang J., Kumar S., Hanna G.B. (2014). Investigation of C3-C10 aldehydes in the exhaled breath of healthy subjects using selected ion flow tube-mass spectrometry (SIFT-MS).. J. Breath Res..

[67] Belluomo I., Boshier P.R., Myridakis A. (2021). Selected ion flow tube mass spectrometry for targeted analysis of volatile organic compounds in human breath.. Nat. Protoc..

[68] Bruderer T., Gaisl T., Gaugg M.T. (2019). On-line analysis of exhaled breath: Focus review.. Chem. Rev..

[69] Wallace M.A.G., Pleil J.D. (2018). Evolution of clinical and environmental health applications of exhaled breath research: Review of methods and instrumentation for gas-phase, condensate, and aerosols.. Anal. Chim. Acta.

[70] Di Gilio A., Palmisani J., Ventrella G. (2020). Breath analysis: Comparison among methodological approaches for breath sampling.. Molecules.

[71] Boots A.W., Bos L.D., van der Schee M.P., van Schooten F.J., Sterk P.J. (2015). Exhaled molecular fingerprinting in diagnosis and monitoring: Validating volatile promises.. Trends Mol. Med..

[72] Hauschild A.C., Schneider T., Pauling J. (2012). Computational methods for metabolomic data analysis of ion mobility spectrometry data-reviewing the state of the art.. Metabolites.

[73] Zemek PG Proton Transfer Reaction Mass Spectrometry (PTRMS) for Ambient and (Compliance) Source Testing Discussion A draft ASTM method is also being developed.

[74] Weraduwage S.M., Rasulov B., Sahu A., Niinemets Ü., Sharkey T.D. (2022). Isoprene measurements to assess plant hydrocarbon emissions and the methylerythritol pathway.. Methods Enzymol..

[75] Zhang X, Frankevich V, Ding J, Ma Y, Chingin K, Chen H (2023). Direct mass spectrometry analysis of exhaled human breath in real‐time.. Mass Spectrom Rev.

[76] Španěl P., Smith D. (2011). Progress in SIFT‐MS: Breath analysis and other applications.. Mass Spectrom. Rev..

[77] Westphal K., Dudzik D., Waszczuk-Jankowska M., Graff B., Narkiewicz K., Markuszewski M.J. (2022). Common strategies and factors affecting off-line breath sampling and volatile organic compounds analysis using thermal desorption-gas chromatography-mass spectrometry (TD-GC-MS).. Metabolites.

[78] Majchrzak T., Wojnowski W., Lubinska-Szczygeł M., Różańska A., Namieśnik J., Dymerski T. (2018). PTR-MS and GC-MS as complementary techniques for analysis of volatiles: A tutorial review.. Anal. Chim. Acta.

[79] Tranchida P.Q., Franchina F.A., Dugo P., Mondello L. (2016). Comprehensive two‐dimensional gas chromatography‐mass spectrometry: Recent evolution and current trends.. Mass Spectrom. Rev..

[80] King J., Koc H., Unterkofler K. (2013). Physiological Modeling for Analysis of Exhaled Breath..

[81] Böhme D.K. (2016). Ion-molecule reactions in mass spectrometry.. Encycl Spectrosc Spectrom.

[82] Marcondes-Braga F.G., Gutz I.G.R., Batista G.L. (2012). Exhaled acetone as a new biomaker of heart failure severity.. Chest.

[83] Samara M.A., Tang W.H.W., Cikach F. (2013). Single exhaled breath metabolomic analysis identifies unique breathprint in patients with acute decompensated heart failure.. J. Am. Coll. Cardiol..

[84] Cikach F.S., Dweik R.A. (2012). Cardiovascular biomarkers in exhaled breath.. Prog. Cardiovasc. Dis..

[85] Risby T. F.T.-O. engineering, undefined 2010, Current status of midinfrared quantum and interband cascade lasers for clinical breath analysis, Spiedigitallibrary. OrgTH Risby, FK TittelOptical Eng. https://www.spiedigitallibrary.org/journals/Optical-Engineering/volume-49/issue-11/111123/Current-status-of-midinfrared-quantum-and-interband-cascade-lasers-for/10.1117/1.3498768.short(accessedSeptember4,2023).

[86] Nardi Agmon I., Broza Y.Y., Alaa G. (2022). Detecting coronary artery disease using exhaled breath analysis.. Cardiology.

[87] Pappas L.K., Giannopoulos G., Loukides S. (2014). Exhaled breath condensate in acute and chronic heart failure: New insights into the role of lung injury and barrier dysfunction.. Am. J. Respir. Crit. Care Med..

[88] Pappas L., Filippatos G. (2011). Pulmonary congestion in acute heart failure: From hemodynamics to lung injury and barrier dysfunction.. Rev. Esp. Cardiol..

[89] Schuster A., Thakur A., Wang Z., Borowski A.G., Thomas J.D., Tang W.H.W. (2012). Increased exhaled nitric oxide levels after exercise in patients with chronic systolic heart failure with pulmonary venous hypertension.. J. Card. Fail..

[90] Witt K, Fischer C, Reulecke S (2013). Electronic nose detects heart failure from exhaled breath.. Biomed Eng / Biomed Tech.

[91] Agostoni P., Bussotti M. (2003). Exhaled nitric oxide and exercise performance in heart failure.. Arch. Physiol. Biochem..

[92] Sola Martínez R.A., Pastor Hernández J.M., Lozano Terol G. (2020). Data preprocessing workflow for exhaled breath analysis by GC/MS using open sources.. Sci. Rep..

[93] Vlasova T. (2015). Organ lipid distress syndrome in the pathogenesis of the progression of surgical endotoxicosis..

[94] Morita T.C.A.B., Trés G.F.S., Criado R.F.J., Sotto M.N., Criado P.R. (2020). Update on vasculitis: an overview and dermatological clues for clinical and histopathological diagnosis - part I.. An. Bras. Dermatol..

[95] Raftery D. (2014). Mass spectrometry in metabolomics: Methods and protocols.. Methods Mol. Biol..

[96] Sutaria S.R., Gori S.S., Morris J.D., Xie Z., Fu X.A., Nantz M.H. (2022). Lipid peroxidation produces a diverse mixture of saturated and unsaturated aldehydes in exhaled breath that can serve as biomarkers of lung cancer—a review.. Metabolites.

[97] Kanoh S., Kobayashi H., Motoyoshi K. (2005). Exhaled ethane.. Chest.

[98] Plantier L., Smolinska A., Fijten R. (2022). The use of exhaled air analysis in discriminating interstitial lung diseases: A pilot study.. Respir. Res..

[99] Knuuti J., Wijns W., Saraste A. (2020). 2019 ESC Guidelines for the diagnosis and management of chronic coronary syndromes.. Eur. Heart J..

[100] McGraw K.E., Riggs D.W., Rai S. (2021). Exposure to volatile organic compounds - acrolein, 1,3-butadiene, and crotonaldehyde - is associated with vascular dysfunction.. Environ. Res..

[101] McGraw K. (2021). Identifying the cardiovascular effects of multiple pollutants..

[102] Sharma R., Zang W., Tabartehfarahani A. (2023). Portable breath-based volatile organic compound monitoring for the detection of COVID-19 During the circulation of the SARS-CoV-2 delta variant and the transition to the SARS-CoV-2 omicron variant.. JAMA Netw. Open.

[103] Ma C.M., Lin L.Y., Chen H.W., Huang L.C., Li J.F., Chuang K.J. (2010). Volatile organic compounds exposure and cardiovascular effects in hair salons.. Occup. Med..

